# Hazards Associated With Nanotechnology in Clinical Dentistry

**DOI:** 10.7759/cureus.46978

**Published:** 2023-10-13

**Authors:** Farheen Tafti, Suyog Savant, Tanvi Saraf, Sujata Pinge, Rohit Thorat, Vivek Sharma

**Affiliations:** 1 Pediatric and Preventive Dentistry, Bharati Vidyapeeth (Deemed-to-Be University) Dental College and Hospital, Navi Mumbai, IND; 2 Public Health Dentistry, Bharati Vidyapeeth (Deemed-to-Be University) Dental College and Hospital, Navi Mumbai, IND; 3 Prosthodontics, Bharati Vidyapeeth (Deemed-to-Be University) Dental College and Hospital, Pune, IND; 4 Periodontics, Bharati Vidyapeeth (Deemed-to-Be University) Dental College and Hospital, Navi Mumbai, IND

**Keywords:** nanotoxicity, dentistry, nanodentistry, hazards, nanomaterials, nanoparticles

## Abstract

Nanotechnology has transformed the field of dentistry with immense potential to provide comprehensive oral health care using nanomaterials, advanced clinical tools, and devices. New materials with superior properties can be developed using nanotechnology by making use of their atomic or molecular properties. Although there are numerous ways in which nanomaterials impact our health, the primary cause is that they comprise chemicals that may have an inadvertent reaction in the body. Moreover, they are used on a daily basis, increasing human contact with them. It is observed to be smaller in size than the physiological barrier in our bodies, making it much simpler for them to pass through and enter the body and they are being used more frequently. It is observed to be smaller in size than the physiological barrier in our bodies, making it much simpler for them to pass through and enter the body and being used more frequently. Although there are numerous ways in which nanomaterials impact our health, the primary cause is they comprise chemicals that may have an inadvertent reaction in the body. The review discusses various types of toxicity, including the cytotoxicity of composites, carbon nanoparticles, silver nanoparticles (SNPs), and quantum dots. It also covers genotoxicity, the effect of nanoparticles on salivary secretion, oral and gastrointestinal mucosa passage of nanoparticles, the tooth surface microenvironment, and interactions with engineered nanomaterials (ENMs). It is concluded that there is scarce information regarding the presence of chemicals that are released from nanoparticles used in dental materials. Nanotechnology is at an infant stage, although it has progressed by leaps and bounds, hailing a new age that provides better treatment modalities in various branches of dentistry. Although the development and application of nanodentistry are of considerable interest, knowledge regarding the possible toxicity of such materials must be meticulously evaluated, and potential benefits must be weighed against the risks to identify potential gaps in the safety assessment. Further research is needed on workplace exposure to nanoparticles in dentistry.

## Introduction and background

Nanotechnology has transformed the ﬁeld of dentistry with immense potential to provide comprehensive oral health care using nanomaterials, advanced clinical tools, and devices [1]. With the advancements in technology and the emergence of nanoscale information, there are new possibilities for replacing oral tissues lost due to disease. These innovations have allowed for a comprehensive approach to tissue replacement in the oral environment [2]. The word nanotechnology was coined by Norio Taniguchi and introduced by Prof. Drexler. The term “nanodentistry” was ﬁrst popularized in 2000 by research scientist Robert Freitas [3]. The generation of nanotechnology is a considerable invention in the ﬁeld of science and led to the progression of nanomedicine (including nanodentistry) [1]. New materials with superior properties can be developed using nanotechnology by making use of their atomic or molecular properties [4].

Although there are numerous ways in which nanomaterials impact our health, the primary cause is they comprise chemicals that may have an inadvertent reaction in the body. Moreover, they are used on a daily basis, increasing human contact with them. It is observed to be smaller in size than the physiological barrier in our bodies, making it much simpler for them to pass through and enter the body and they are being used more frequently. Thus interrelationship between the nanomaterials, the environment, and human beings is unavoidable (as well as animals, plants, and other living things in the environment) [5].

Nanomaterials are made of highly active materials because of which they can interact and bring about detrimental effects. Once the nanomaterials have entered the body the effects need to be observed as it is an area of concern in the current era [5]. Society of Toxicology has described nanotoxicology as the study of the adverse effects of engineered nanomaterials (ENMs) on living organisms and ecosystems, including the prevention and amelioration of such adverse effects [6,7]. The wide range of the use of nanomaterials in the food sector, medical applications, consumer and pharmaceutical sector, etc., widens the intake through contact with the skin, inhalation, and the oral cavity. The organs or the tissue that were put at risk coincidentally by the nanoparticles are known as "non-targeted organs" or "non-targeted tissue," such interaction often takes place in the oral cavity [5].

## Review

Cytotoxicity of nanocomposite

An amalgamation of nanoparticles within the resinous matrix of the composite has shown satisfactory results as an anticaries agent as well and it has antibacterial properties [8,9]. However, studies have observed discoloration of the composite matrix and there is some concern regarding their biocompatibility [8,10,11]. Copper and zinc-based nanoparticles also produced severe toxic effects in animal studies in vitro [8,12,13]. Studies done by Collard et al. concluded that when there was the removal of an old composite restoration or shaping and polishing of a new composite restoration the dental personnel may inhale aerosolized composite dust [14,15]. Schmalz and Arenholt Bindslev have recommended avoiding inhalation of composite dust [16]. Due to nanoparticles' great mobility, substantial lung penetration, and considerable active surface area, there have been multiple discussions about their alleged detrimental effects on health [9,16]. In particular, reshaping and contouring of composites with rough polishing discs (Sof-Lex discs; Neuss, Germany: 3M) and with a diamond bur resulted in peak concentrations between 0.1 cm-3 and 106 cm-3 in the breathing zone of the dentist. The clinical assessment revealed clearly distinguishable peak moments of high concentrations of nanoparticles in the breathing zone of the dentist and the patient in association with abrasive procedures of composite [9].

Nanoparticles are associated with various adverse effects depending upon their distinct characteristics [16]. They have genotoxic effects, elevated reactive oxygen species (ROS) levels, size and dose-dependent cytotoxicity, cellular uptake, and pulmonary inflammation [17]. These nanoparticles penetrate easily into the lungs and have adverse health consequences that are only localized. They may also translocate into the blood or enter the brain through the olfactory epithelium [18]. Methylmethacrylate is detected in the discharged particles and a few people may be allergic leading to allergic contact dermatitis [9,19].

A recent study was conducted and concluded that the shelf life of composite dental fillings ranged around five years. The physicochemical properties of nanoparticles in loose form lead to the decrease in cellular absorption, and displacing the intracellular location [2].

In another study, it was observed that the direct interaction of nanoparticles with cell membranes does not occur in vivo but through the formation of nanoparticle protein complexes known as "protein coronas" (PC). Nanoparticles discharged into body ﬂuids intercommunicated with their biological components, predominantly protein that accumulated into complexities of protein attached to the nanoparticle surface, which may display significant variations in biodistribution, clearance, activity, and toxicity. The organization of protein coronas had detrimental effects as they helped the physicochemical characteristics but caused the destruction of protein on the other hand [20].

Toxicity of carbon nanoparticles in humans

Dentistry researchers have focused on studying carbon nanoparticles due to their superior properties which include high mechanical strength, acceptable biocompatibility, and high absorption. The impact of carbon nanoparticles is similar to that of steel, which is used to increase the hardness of composites, cements, and concrete. Therefore, carbon nanoparticles are ideal for making various dental materials as they exhibit high tensile strength and stiffness. In addition to being used to reinforce dental materials, carbon nanoparticles have also been found to be suitable as a synthesis material for scaffolds and targeted drug delivery [21].

American Heart Association's scientific statement stated that short-term exposure to elevated particulate matter of carbon nanoparticles in the outdoor air remarkably imparts to increased acute cardiovascular mortality, particularly in some population at-risk subgroups. The risk of succumbing to coronary heart disease is enhanced with prolonged exposure to air pollution. Respiratory exposure may trigger inflammatory reactions, the release of cytokines that stimulate clotting into the bloodstream with potential cardiovascular impacts. The inflammatory nature of particulate matter has been definitively verified, and there is a developing recognition of the adverse effects of particulates on endothelial function, fibrinolysis, and thrombogenesis [22].

Cases have been reported of acute vascular and cardiac dysfunction as an outcome of elemental respiratory exposure. The observed effects on cardiovascular function could be secondary to carbon nanoparticles' direct or indirect actions. Since there are several unsolved scenarios regarding nanoparticles and their potential health effects, constant monitoring and extreme preventive measures have become crucial for individuals who have been exposed. Nanoparticles exhibit the capability to navigate deep into the respiratory tract. Once in the alveolar region, they are able to translocate to the blood and to places far from their original site such as the liver, spleen, kidney, and brain. The migration of distant places is an important concern given the extent of their toxicity. As a result of its high blood supply and inclination to concentrate toxins, the kidney is extremely vulnerable to xenobiotics [23].

Toxicity of silver nanoparticles (SNPs) and quantum dots on mammalian cells

Within the realm of metallic nanoparticles, silver nanoparticles have become a topic of interest in scientific research due to their ability to exhibit biological activity against fungi, bacteria, and enveloped viruses as well as having antimicrobial properties. The mode of action associated with silver nanoparticles is primarily linked with the release of cationic silver and its oxidative potential. Additionally, the synthesis of silver nanoparticles and their size and shape can also play a role in their mechanism of action. Due to their antimicrobial properties and benefits, silver nanoparticles have shown potential as a compound to be used in dentistry. The incorporation of antimicrobial substances in dental biomaterials is a strategy that has been adopted with interest [24].

Quantum dots are known for their ability to emit bright light when exposed to ultraviolet light. They can be coated with a material that allows them to attach specifically to the molecule that needs to be tracked. In the field of cancer research, quantum dots have been used to bind themselves to proteins that are unique to cancer cells, helping to detect and analyze tumors more accurately. This innovative technology has shown great potential in bringing tumors to light and advancing cancer diagnosis and treatment [25].

The foremost theory was the increase in the silver ions released from the cell membrane tweaking its permeability and promoting mortality by oxidative damage, which is how most nanoparticles surface, predominantly through "burst release" [26]. Park et al. described yet another toxicity mechanism of a study conducted in order to assess the detrimental impact of various silver nanoparticles on mouse peritoneal mammalian cell lines. In the study, it was observed that nanoparticles undergo ionization after entering the cell via endocytosis and thereafter release ions that have an influence on the cells from the inside. The "Trojan horse" effect refers to this technique. A drop in the intracellular guanosine triphosphate cyclohydrolase I level, a rise in nitrous oxide level, variations in cell cycle, and change in the protein level and gene expression of tumor necrosis factor (TNF) and matrix metalloproteinases (MMPs) may all result from the co-exposure of cells with silver nanoparticles [2,27]. According to Hsiao et al. "Trojan horse" was very likely to be one of the mechanisms of nanoparticles toxicity to mammalian cells [28]. These two cell-ion interactions were regarded as occurring. First, outside the cell in the early period after exposure and by burst release and later inside the cell in the Trojan horse mechanism [29]. The antibacterial activity was observed in a study by Geng et al. that the incorporation of nanoparticles causes the destruction of soft and hard tissues [30].

A minimum of one report of argyria in humans after chronic ingestion of colloidal silver solution implied the ability to absorb silver from nanosilver [31]. In another study, it was observed that renal damage is by the mechanism of apoptotic impairment and necrotic cell death after long-term oral administration of silver nanoparticles [32].

At the cellular level, cadmium promotes the synthesis of reactive oxygen species (ROS) by depletion of cellular antioxidants. Cadmium alters the mitochondria, triggering apoptosis, which impairs intracellular calcium signaling, breaks deoxyribonucleic acid, and prevents its repair. These distinct features of quantum dots-induced cellular toxicity which strengthen the theory that quantum dots are hazardous. Cadmium ions are gradually released into the cells after the internalization of quantum dots, predominantly as an outcome of imperfections in nanoparticle coatings. Small particle sizes induce an upsurge in the surface volume ratio, which uncovers more cadmium ions thereby rendering them more accessible to negatively impact the environment. At the genomic level the nanomolar quantum dot concentrations, shown to lead to deoxyribonucleic acid perturbations, further lead to quantum dot-induced abnormalities at the genomic level [33]. Cadmium telluride quantum dots when compared to 17-β-estradiol showed the same or higher level of induced cellular proliferation, estradiol receptor R activation, and estrogen-associated rapid nongenomic signaling events. Green (smaller) quantum dots generated a more resilient estrogenic effect than orange (larger) quantum dots. It can be anticipated that quantum dots may be withheld in the lungs and can probably transport into the central nervous system, one speculation is made based on toxicological studies of airborne nanoparticles. It can be anticipated that during quantum dot manufacture or its use in the laboratory, accidental absorption through the skin and eyes may occur. Studies done on models have shown that quantum dots perforate the porcine skin and cause impairing effects, even in low doses [34,35].

Oral and gastrointestinal mucosa passage of the nanoparticles

The excess effusion of destructive ions in the oral cavity and gastrointestinal tract depends on the dissolution of different nanoparticles. There is scarce literature available to unveil the interlinkage between the given nanoparticle and different parts of the gastrointestinal tract [2].

Gaillet and Rouanet conducted a review and established that orally administered and absorbed nanoparticles can be probably found in the lungs, bone marrow, brain, pelt, eyes, bladder, stomach and teeth, tongue, salivary glands, thyroid, and parathyroid [36]. Despite being the most widely recognized model for drug delivery/pharmaceutical investigations, the mechanism of action of nanoparticles on the buccal mucosa is still unknown. This is probably a result of extensive nanoparticle interactions in the oral cavity, which are impossible to duplicate in vitro [37]. In their research, Teubl et al. observed that electrostatic repulsion induced a decline in the particle-cell membrane contact and cellular uptake of single nanoparticles, which resulted in their infiltration and penetration of only the upper portions of the epithelium [38]. The primary size of the nanoparticles is crucial to their cellular absorption in the buccal mucosa. As the intubation of the buccal mucosa can take up to 14 days, nanoparticles continue to reside in the buccal mucosa before moving further to the gastrointestinal tract through the esophagus to the stomach [37]. The physicochemical properties of the nanoparticles were re-organized, as the nanoparticles interrelate with the gastric juices in the stomach. The chances were high of the nanoparticles reaching the intestine after getting through the severe conditions of gastrointestinal digestion [39]. Nanoparticles >200 nm get deposited and captured in the mucus layer submucosa or gut-associated lymphoid tissue of the small intestine. The mechanism for cellular uptake which was seen in the small intestine is endocytosis. Bergin and Witzmann observed nanoparticles were present in the liver and brains of the rodents after oral exposure. The oral cavity, head, and neck region have a high degree of vascularization, which is a characteristic found throughout the human body. This has led to the consideration of blood as a likely route for certain medical treatments and interventions in these areas [40,41].

Genotoxicity

The use of nanotechnology in the field of dentistry is restricted due to its effects on genotoxicity. It could trigger uncontrolled cell proliferation and cause genetic material alterations for all time. Genotoxicity can be classified as primary or secondary genotoxicity. They are termed direct effect and indirect effect mechanisms. The concept of "primary genotoxicity" emphasizes the manifestation of genetic damage without any inflammatory response. The approaches that encompass the particle’s interaction directly with the genetic material and associated proteins elicit the direct primary effects. These inflammatory responses attract these cells into the course of innate immunity; however, free radicals are formed during this process, which ultimately leads to deoxyribose nucleic acid damage. Three primary hypotheses pertaining to the genotoxic mechanism have been extensively investigated by researchers namely the involvement of the surface effect, the release of reactive oxygen species due to the action of released transition metal ions by nanoparticles, and the activation of membrane receptors like epidermal growth factor receptor by transition metals. The various effects of genotoxicity observed are impact on deoxyribose nucleic acid damage, impact on deoxyribose nucleic acid repair processes, i.e., sequestration of deoxyribose nucleic acid repair proteins in nanoparticle - protein corona, and impact on deoxyribose nucleic acid repair protein. Another is the impact on the epigenome, i.e., effect on histone modification, effect on non-coding ribonucleic acid, and effect on deoxyribose nucleic acid methylation [42]. According to Sujatha et al. in 2011, exposure to nanomaterials resulted in genetic abnormalities, such as chromosomal mutations, deoxyribose nucleic acid fragmentation, and modifications in gene expression profiles [43]. Deoxyribose nucleic acid damage occurred as a consequence of both direct and indirect mechanisms when the nanomaterials approached the human body via the lungs, skin, or oral pathways. Nanoparticles have been observed to cross the cell membrane and interact with the nucleus during mitosis, leading to the division of the cell into daughter cells. This interaction involves direct contact with the cell's genetic material, which can result in interference with deoxyribose nucleic acid. The quantum dots penetrated the nucleus membrane after achieving their target and binding with histone proteins [44]. The significant intracellular production of reactive oxygen species and low levels of antioxidants were the primary contributors to the genotoxicity of nanomaterials. Nanomaterials enhanced corrosion, which unleashed metal ions and chronic inflammatory reactions. This led to the generation of advanced oxidative deoxyribose nucleic acid damage, particular signaling pathways including mitogen-activated protein kinase (MAPK), and NF-kB activated by oxidative stress, which along with the reduction of antioxidant defenses, advanced the release of pro-inflammatory cytokines, trigger inflammation, by reactive oxygen species release from inflammatory cells (e.g., neutrophils) [45].

Effect on salivary secretion

The vast majority of saliva’s components - water, ions, and proteins - are actively released by salivary glands in the mouth. The bulk of the functionally significant protein components of saliva are produced and secreted by acinar cells, resulting in the formation of acinar secretory units which collectively make up the secretary endpiece of the salivary glands. As salivary gland secretion is a nerve-mediated reflex, it ceases nearly completely, in particular, it is compromised. Excitation of sympathetic nerves tends to increase the levels of protein content, bringing about the formation of viscous saliva. Cholinergic parasympathetic innervation of the salivary glands regulated salivary secretion, which stimulated the release of saliva from the acinar cells [46]. Whenever high milligram doses of engineered nanomaterials were introduced, specifically to an isolated nerve preparation, the capacity of peripheral nerves to generate an action potential was unaltered [47]. However, the response of engineered nanomaterials to the secretory activity of salivary glands has not been studied yet. Lysozyme adsorbed onto the surface of some engineered nanomaterials resulted in an alteration of the spatial arrangements of the β-sheets in the enzyme tertiary structure which resulted in the loss of its antibacterial properties. As a result of lysozyme adsorption onto the surface of certain engineered nanomaterials, such as TiO2, the spatial arrangements of the β-sheets within the enzyme's tertiary structure were altered, leading to the loss of its antibacterial properties. It is important to note that the main cause of this phenomenon was the adsorption of lysozyme onto the surface of these engineered nanomaterials [32].

The tooth surface microenvironment and interactions with engineered nanomaterials

Salivary substances lack the ability to interact directly with tooth enamel, and instead interact by means of a thin film known as the pellicle, which generally encases the entire dentition. The pellicle is an acellular proteinaceous film of salivary origin that tends to form in minutes and is firmly bonded to the enamel. It is formed by saliva. The principal components of saliva are salivary glycoproteins, phosphoproteins, lipids, and to a lesser extent, components from the gingival crevicular fluid. It passively governs the transport of ions in and out of the dental tissue, the pellicle plays an integral part in the processes of tooth demineralization and remineralization. The pellicle a selective semi-permeable structure that functions as a chemical buffering barrier suppresses bacterial and dietary acid demineralization of the enamel’s mineral content. However, the newly formed pellicle might not have the ability to successfully prevent engineered nanomaterials penetration [32]. There are a few engineered nanomaterials that are small as they are theoretically infiltrated into the porous mineral structure of the enamel-pellicle. The biofilm that forms on the pellicle acts as an important obstacle to engineered nanomaterial diffusion at the interface (above), withering through steric hindrance (e.g., engineered nanomaterials becoming trapped or entangled with the biofilm protein matrix) or by adsorption of the engineered nanomaterial onto the glycoprotein coat of the S-layer of microbes [48]. The process of protein corona formation is bound to be dynamic, with bacteria releasing proteinaceous material as the biofilm thickens and bulk fluid shifts into the pellicle permitting some adsorption into the film. Understanding the engineered nanomaterials encased in salivary corona communes with the biofilm and the bacteria within it, on the other hand, there is a crucial gap in determining oral engineered nanomaterials bioavailability. Further investigation is necessary to ascertain the details of how oral-engineered nanomaterials adhere to the dental surface and the potentially complex interactions between the engineered nanomaterials and the bacteria and their secretory products, specifically the proteins that make up the underlying pellicle layer [32].

## Conclusions

There is scarce information regarding the presence of chemicals that are released from the nanoparticles used in dental materials. Very limited data exists regarding the inhalation of very fine respirable particles on health. The methods characterizing exposure and translocation of nanoparticles in the body are still in the infant stage and there are reports of nanoparticles clearing from airways readily and gaining access to circulation. Studies have also substantiated the interaction of the nanomaterials with respect to the attached gingiva and the alveolar bone or alveolar mucosa. Nanotechnology has made significant progress, but unfortunately, its success has been limited by the negative impact it can have on non-targeted organs. As it is in the infancy stage, its interaction with the non-targetted organs of the human body is unknown due to the limited experimental procedures being performed. Evaluation of the cytotoxicity of the materials and their potential usage is questionable due to the lack of knowledge which has to be ardently explored. There is a need to explore the pros and cons of nanomaterials and the safety standards need to be set so as to have a symbiotic relationship with the human body. Further research is needed on workplace exposure to nanoparticles in dentistry. The data so far indicates that oral toxicity for nanoparticles is low, but some nanoparticles are translocated across the gut to cause systemic disturbances, perhaps with organ pathology.
